# Person-centred crisis support services as alternatives to emergency departments: a systematic scoping review

**DOI:** 10.1007/s00127-024-02776-1

**Published:** 2024-10-10

**Authors:** Danielle Postorivo, Stephen Parker, Harvey Whiteford, Zoe Papinczak, Zoe Rutherford

**Affiliations:** 1 School of Public Health, Faculty of Medicine, University of Queensland, Brisbane, QLD Australia; 2 Queensland Centre for Mental Health Research, Brisbane, QLD Australia; 3 Institute for Health Metrics and Evaluation, University of Washington, Seattle, WA USA; 4 Metro North Mental Health, Brisbane, QLD Australia; 5 School of Human Movement and Nutrition Sciences, The University of Queensland, Brisbane, QLD Australia

**Keywords:** Mental Health Crisis, Crisis Support services, Person-centred, Alternatives to Emergency Departments

## Abstract

**Purpose:**

To identify, critically appraise, and synthesise the published and grey literature on person-centred crisis support services as an alternative to support in emergency departments (EDs) for people experiencing mental health crises. This scoping review explores the characteristics and outcomes of these services.

**Methods:**

A systematic scoping review was undertaken to identify publications describing person-centred crisis support services and their outcomes. Search strings were applied to multiple databases, and publications were subjected to quality appraisal. The review process was informed by The Joanna Briggs Institute (JBI) Manual for Evidence Synthesis and the Preferred Reporting Items for Systematic Reviews and Meta-Analysis Extension for Scoping Reviews (PRISMA-ScR).

**Results:**

Thirteen publications were included in the narrative synthesis, and these considered eight separate crisis support services. The methodological quality of the publications included was limited. Key findings were positive visitors’ experiences, high rates of ED deflection, and overlaps between repeat visits, crises prevention, and hospital avoidance. Key recommendations included increasing opening hours and capacity and improving service awareness and accessibility.

**Conclusions:**

The available evidence suggests that person-centred crisis support services are perceived by stakeholders as safe and effective alternatives to EDs for people experiencing mental health crises, providing more timely and appropriate care while reducing ED mental health presentations. Due to the limited quality of the publications included, high-quality research is needed to better understand the model and confirm the findings reported in this review.

**Supplementary Information:**

The online version contains supplementary material available at 10.1007/s00127-024-02776-1.

## Introduction

Emergency departments (EDs) are the default assessment and support context for people in need of mental health crises^1^ care worldwide [1–3]. However, it has been widely acknowledged that EDs are not ideal settings for people experiencing these crises, as they often report that the ED environment is overstimulating and intimidating and that staff do not respond appropriately, resulting in experiences of humiliation, discrimination and trauma [1–6]. Additionally, EDs have been experiencing rising numbers of mental health presentations worldwide, and individuals presenting with mental health crises often experience longer wait times than physical health presentations [1, 3]. The lengthy waits and the tension of the ED environment are associated with poorer clinical outcomes, including increased patient mortality and increased workload for staff [3, 4, 7, 8].

Some people presenting to EDs with mental health crises do not require urgent clinical care but feel that the ED is their only avenue for immediate support due to a lack of alternatives [8]. People with lived experience of mental health crises and mental health care professionals have called for person-centred alternatives to EDs, which provides an opportunity to deflect people from a busy ED into a more suitable environment for mental health care [3, 4]. Person-centred care is health care that is respectful of, and responsive to, the preferences, needs and values of the person receiving care [9]. Therefore, to establish mutual trust and respect, the person-centred approach aims to engage with the individual’s personal experiences, as well as their families, caregivers, and support network, rather than only addressing their medical condition [9]. This contrasts with the ED setting, where care is disease-focused, and staff in a busy and stressful environment often prioritise medical-technical interventions with a focus on maintaining patient throughput [10].

According to the World Health Organization, person-centred crisis support services should provide visitors with adequate care and support in a safe space and comfortable environment without the use of force or coercion and that respect the right to legal capacity and other human rights, understanding that people experiencing crises are experts when it comes to their own care and support needs [11]. However, the literature on services that have implemented this approach is scarce. There are currently no reviews of the published (peer-reviewed) and unpublished (grey) literature considering person-centred crisis support services as alternatives to EDs. Therefore, this scoping review aimed to analyse the published and unpublished literature on person-centred crisis support services to better understand how they function as alternatives to EDs for people experiencing mental health crises and to inform future research.

## Review question

What is known from the existing literature about person-centred crisis support services as alternatives to EDs?

### Additional questions

- What are the characteristics of person-centred crisis support services?
- What are the outcomes of person-centred crisis support services as alternatives to EDs for people experiencing mental health crises?

## Methods

### Protocol and registration

This review followed the Preferred Reporting Items for Systematic Reviews and Meta-Analysis Extension for Scoping Reviews (PRISMA-ScR) checklist [12] as a reporting guideline (Supplementary material, File 1, Online Resource 1) complemented by The Joanna Briggs Institute (JBI) Manual for Evidence Synthesis [13]. The review protocol was registered prior to commencement (10.17605/OSF.IO/M2U8C).

### Search strategy

Information sources included peer-reviewed and grey literature. Search strings and associated databases Google, Google Scholar, PubMed and Scopus were determined in consultation with a health research librarian. The search strategy used a combination of terms related to person-centred crisis support services, alternatives to EDs and service evaluation. All search terms are shown in the supplementary material (File 1, Online Resource 2).

### Study selection

One author (DP) applied the eligibility criteria to title and abstract screening on the search results for each database. Duplicates were removed by importing the selected references from each database directly into Endnote 20 (grey literature references were added manually). Full texts were screened by two authors (DP and ZP) and removed according to eligibility criteria, with disagreement between the reviewers resolved through discussion and consensus.

### Inclusion criteria

Published and grey literature were included if they were written in English and focused on describing and/or evaluating person-centred mental health crisis support services as alternatives to EDs. Additional articles containing supplementary information on the selected publications were also eligible (e.g. evaluation frameworks).

### Exclusion criteria

Non-acute crisis support models, such as residential and day programs were excluded. Furthermore, interventions not offered in a physical space were excluded (e.g. telephone crisis support).

### Evidence synthesis

The data was extracted and categorised by one author (DP), based on the research questions agreed upon by all authors during the development of the review protocol. The categorised information, aligned with the research questions, aimed to display the characteristics and outcomes of the person-centred crisis support services retrieved from the publications included. These findings were compiled in the results section and summarised in Table 1 (characteristics), 2, 3, and 4 (outcomes) by one author (DP), after multiple meetings and thorough discussions among all authors.

## Results

### Search and selection of records

**Fig. 1:**
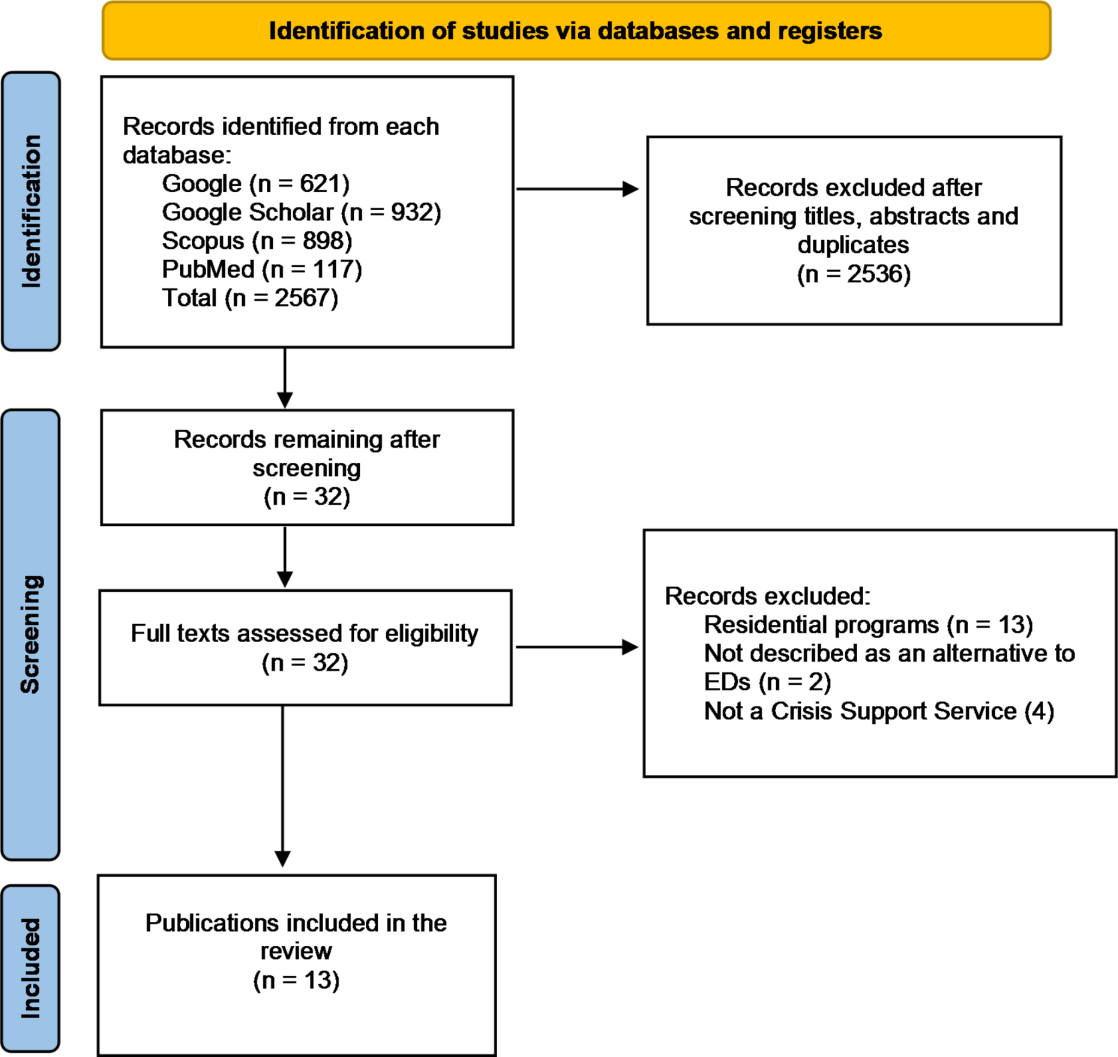
Flow diagram of data extraction

Thirteen records were identified for inclusion through the systematic search process (Fig. 1).

### Overall description of publications

Thirteen publications were included [14–26], and eight services were identified. This included five peer-reviewed articles [14, 17, 23, 24, 26], one evaluation framework [25], and seven grey literature reports [15, 16, 18–22]. From the thirteen publications, nine provided information about service characteristics [14–22] and twelve provided information on service outcomes (two were specifically focused on visitors’ experiences) [14–24, 26].

### Quality appraisal

All publications presenting data on each of the services included were subjected to quality appraisal [14–24, 26]. Complete appraisals and criteria explanation are provided in supplementary material (File 2). Two authors (DP and SP) completed the appraisals using the relevant JBI checklists (economic evaluations, qualitative research, and text and opinion) [27–29]. Disagreements were resolved via collaborative discussion.

The methodological quality must be considered when drawing conclusions about person-centred crisis support services from the publications provided. None of the publications were assessed as high-quality; three were fair-quality (two peer-reviewed qualitative studies and one grey literature report) [17, 19, 23], and, therefore, most of the publications included were rated as poor quality for the purposes of this review [14–16, 18, 20–22, 24–26]. Most common issues included limited description of methodology, rudimentary cost-effectiveness analyses, and inadequacy of methods such as survey methodology for interpretation of qualitative outcomes and trend-based assessments for quantitative and qualitative outcomes.

### Characteristics of person-centred crisis support services

From the nine publications describing service characteristics [14–22], the main aspects identified in all services were that they aim to offer one-to-one support in a safer environment than EDs to people experiencing mental health crises where they may feel listened to and respected, avoid long waits and be connected to other community services (see Table 1). Key features included the leadership from peer workers with lived experience of mental health crises, the non-medical, calming, and relaxing environment, and the option of self-presenting to the services, which was considered an important aspect for avoiding EDs and long waiting periods. Only one service seems to differ in some of these core features, The Crisis Hubs [18], as they do not mention the lived experience workforce or a non-medical environment and have a team of medical staff. Three of the services are described as “Crisis Cafés” or “Safe Haven Cafés” [15, 16, 20, 21] but there are no specific differences from the other person-centred crisis intervention services mentioned, except that they are designed to resemble cafés. Two services are hospital-based [18, 21], while all others are community-based; no other differences were indicated between the two types of services.

In the services with peer workers, these staff are responsible for most of the support provided, including the one-to-one chats and working collaboratively with the visitors to develop their coping skills and safety planning. Clinicians provide support with clinical skills and external referrals when required. Although all services were presented with a general description, only one provided a comprehensive description of staffing roles and training procedures, as well as the physical space (where they discuss the choice of non-clinical furniture to compose a non-medical environment), and therefore, the only one to describe the layout of a non-medical environment [14]. A more thorough description of these aspects from the other services would be essential to better understand how they function.

**Table 1:** Characteristics of identified Crisis Support services

Service			Provided by the service																							
			One-to-one support		Self-management/ Coping skills			Safety Plan			Group support		Telephone support			Non-medical environment		Food and drinks		Transportation			External referrals		Other amenities	
Aldershot Safe Haven Café [18, 19]			✔		✔			✔			-		-			✔		✔		-			✔			
Community Crisis Intervention Service [20]			✔		✔			✔			-		-			✔		✔		-			✔			
Crisis Hubs [21]			✔		?			?			-		-			?		✔		-			✔			
Dial House [22]			✔		✔			✔			✔		✔			✔		✔		✔			✔		Bath, computers with internet access, and family room to enable parents in crisis to access the service.	
Hillingdon Cove Crisis Café [23]			✔		✔			✔			-		✔			✔		✔		-			✔			
St Vincent Safe Haven Café [24]			✔		✔			?			-		-			✔		✔		-			✔		Space to rest/take a nap	
Safe Spaces [25]			✔		✔			✔			-		-			✔		?		-			✔			
The Living Room [17]			✔		✔			✔			-		-			✔		✔		✔			✔		Bath and healthy food, separate space to rest	
Service	Country			First year		Model				Location					Staffing							Operational Hours			Access	
						Crisis Café	Crisis Intervention			Community		Hospital			Lived experience peer workers		Mental health clinicians		Volunteers			After hours (some include afternoon)	24 h		Drop in	Referrals from hospital and community
Aldershot Safe Haven Café [18, 19]	England			2014		✔				✔					✔		✔		-			✔			✔	✔
Community Crisis Intervention Service [20]	Northern Ireland			2019			✔			✔					✔		✔		-			✔			✔	✔
Crisis Hubs [21]	England			2020			✔					✔			?		✔		-				✔		✔	✔
Dial House [22]	England			1999			✔			✔					✔		✔		✔			✔			✔	✔
Hillingdon Cove Crisis Café [23]	England			2021		✔				✔					✔		✔		-			✔			✔	✔
St Vincent Safe Haven Café [24]	Australia			2018		✔						✔			✔		✔		-			✔			✔	✔
Safe Spaces [25]	Australia			2022			✔			✔					✔		✔		-			✔			✔	✔
The Living Room [17]	USA			2011			✔			✔					✔		✔		-			✔			✔	✔

### Outcomes of crisis support services

Twelve publications displayed information on service outcomes [14–24, 26]. Findings from all twelve publications, alongside study design, methods and quality assessment are detailed in Table 2, and a summary of key findings is displayed in Table 3.

#### Stakeholders’ perspective

As illustrated in Tables 2 and 3, all services that reported on visitors’ experiences were considered safe and effective. Other key findings were that visitors reported that they were treated with dignity and respect, had a better experience than at EDs and avoided long wait times by visiting the crisis support services instead of the EDs [15–19, 21–24].

In the only two studies that focused specifically on visitors’ experiences (two fair-quality studies according to quality appraisal), participants reported that the services provided feelings of connectedness and being cared for without judgment and discrimination, where they can go for help and respite during a crisis. In contrast, their ED experiences were characterised by feelings of insecurity, loneliness, intimidation and stigma, which may exacerbate a crisis rather than resolve it [17, 23]. Some of the positive aspects of the crisis support services also reported in these two studies were the non-medical environment, the interactions with non-judgemental staff, the use of lay communication and the provision of a cup of tea and a blanket for warmth, as well as the space to speak at their own pace with no time restrictions [17, 23].

Regarding other stakeholders’ perspectives, only three studies interviewed staff [18, 19, 23]. Overall, staff had the same thoughts of the services as reported by visitors in that they are comfortable, safe and non-judgmental spaces and that they felt valued and supported as staff [18, 19, 23]. Broader stakeholders’ perspectives were only illustrated by the Crisis Hubs [18]. Commissioners, community mental health teams, and police and ambulance services highlighted that the service offers timely care, diverting visitors from lengthy ED waits to more appropriate mental health support, as well as relieving ED staff’s burden. Regarding the ED staff perspective, they reported that it is unclear whether ED psychiatric liaison services or the Crisis Hubs are the safest and most person-centred pathway for visitors.

#### Repeat visits, crises prevention and hospital avoidance

Another key finding was the significant rates of repeat visits in some of the services. This is an important finding due to repeat visitors not being explicitly addressed as part of the target group of crisis support services. In Aldershot Safe Haven, 97% of survey respondents said they visited the service on more than one occasion, and 92% visited more than four times [15]. Over a third of visitors of the Crisis Hubs were repeat visitors [18]. While Dial House does not display a specific number for all repeat visitors, it reported that over 50% of all visitors had visited the service up to 3 times, while 30% made a few visits in most years, 12.5% used it extensively in one year, and 7.5% became long-term frequent users [19]. In the Safe Spaces, 32% of visitors were repeat visitors, and repeat visits had increased over the months to 90% of all visits (from 72 to 90% in 6 months) which means that most of service activity is directed to repeat visitors [22]. The Living Room’s qualitative study [23] mentioned that interviewees visited the service up to 20 times but did not specify how many times per participant.

In terms of the impacts of these findings, there was an association between repeat visits, crises prevention and hospital avoidance across these publications. In Aldershot Safe Haven, survey results showed that almost all visitors are repeat visitors and that most people present to prevent a crisis rather than in a crisis [15]. Additionally, 18% of visitors stated that Aldershot Safe Haven had helped them the most with ‘staying alive,’ suggesting suicidal crisis prevention. Dial House’s study [19] reported that the service helped prevent long-term visitors from getting worse by helping them maintain their coping strategies and progress to greater recovery. It’s also noted in this study that Dial House has drawn its success stories mainly from its longer-term users: individuals for whom, according to the study, it has provided a route from the verge of suicide to recovery through volunteering and eventually to paid employment. The other article on Dial House [24] reinforces that the service helped visitors avoid ED and prevent crises and that up to 75% of visitors to Dial House are suicidal. In the Community Crisis Intervention Service study [17], although data on repeat visitors was not provided, visitors reported the importance of early help-seeking as a strategy against suicide and hospital avoidance, and that they felt comfortable seeking help at the service at an early enough point to prevent a situation from escalating to the point where there is self-injury and the need for hospital care. In the Crisis Hubs report [18], it was reported that repeat visitors of the hubs are more likely to go directly to a hub rather than to an ED, which reduces the number of ED mental health presentations, according to the study. In the Safe Spaces report [22], repeat visits were also associated with hospital avoidance, as according to the report allowing visitors to re-visit has enabled the avoidance of 176 visits to EDs [22].

Additionally, it was reported in the Aldershot Safe Haven and St Vincent Safe Haven evaluations that the main reason for visitors to attend the services was socialising with people like them [16, 21]. From the first evaluation of Aldershot Safe Haven to the second, this answer changed from socialising to maintaining well-being during a difficult time [15]. However, socialising was still reported as the main activity at the café. Considering that most visitors in Aldershot are also repeat visitors who attend to prevent crisis and most people in both services also reported that their crisis might have escalated if the services weren’t available, these results may indicate an overlap between social connectedness, maintaining well-being and crises prevention, which can also be associated with repeat visits and hospital avoidance as mentioned before.

**Table 2:** Publications, methods and findings

Service	Authors	Design	Methods	Quality Appraisal	Key Findings
Aldershot Safe Haven	Surrey and Borders Partnership Foundation NHS Trust, 2014 [19]	Service evaluation	Utilised visitor feedback survey provided by the service and ED data	Poor	• 99.49% of visitors reported that they were always treated with dignity and respect • Most people (38%) reported socialising as the main reason for visiting • Self-reporting suggests that there have been 63 people using the Safe Haven as an alternative to ED • Most people (38%) reported that if the service wasn’t open, it may have escalated their crises
Aldershot Safe Haven	Griffiths & Gale, 2017 [18]	Service evaluation	Utilised visitor feedback survey provided by the service and ED data	Poor	• 97% of visitors said they felt treated with dignity and respect • 97% of respondents have visited the Safe Haven on more than one occasion, and 92% have visited the service more than four times • 13% of people attended in crisis, while 56% attended to prevent themselves from escalating into a crisis • 53% of people who had attended ED before Safe Haven showed a decrease in ED attendance following their introduction to the service
Community Crisis Intervention Service	Ennis & Walker, 2022 [20]	Peer-reviewed qualitative study on visitors’ experiences	Interviews with visitors and quantitative data on visitors provided by the service	Fair	• Those interviewed found the service empathetic and successful in de-escalating their state of crisis. It was perceived as non-judgemental and compassionate • Interviewees discussed the service as a place where they felt comfortable seeking help at an early enough point to prevent a situation from escalating to the point where there is self-injury and the need for hospital care • ED brought connotations such as drama, fear, stigma and long waiting times. This was considered something that may exacerbate a crisis rather than resolve it • The brief psychological interventions within the service were sufficient to de-escalate the crisis for many (*n* = 117). Some others only required additional signposting to other non-emergency services (*n* = 17)
Crisis Hubs	Health Innovation Network, 2022 [21]	Service evaluation	Interviews with broader stakeholders, staff and visitors; visitor feedback survey provided by service	Poor	• Most visitors across sites (56%) said they felt safe at a Crisis Hub. However, a third, 33%, did not feel safe. • 36% of visitors in the survey felt they received the support and treatment needed, but around half (51%) thought they did not. However, from the interviews, the majority of visitors said they felt safe and were treated with kindness, dignity and respect • 51% felt listened to by staff compared to 37% who did not. Interviews with visitors revealed varied experiences that highlighted that some staff could benefit from training in listening skills to improve the quality of person-centred care • The majority of staff were satisfied with their workplace, training, senior leadership and reported feeling treated with dignity and respect by their colleagues • The evaluation highlighted that the strengths of the Crisis Hubs approach, particularly the two hubs that provide direct access to people in a mental health crisis (not via an ED) are timeliness, length of stay, and repeat access for visitors
Crisis Hubs	The Health Innovation Network & UCL Partners, 2020 [28]	Evaluation framework	Consultations with stakeholders involved in the service to develop an evaluation framework specific to the Crisis Hubs	N/A	• The evaluation framework utilised to evaluate the Crisis Hubs and generate the findings above was developed from pre-workshop interviews with clinical and/or service leads from 8 of the Mental Health Trusts and other stakeholders with expertise in mental health crisis pathways; a workshop inviting a wide range of stakeholders including Mental Health Trust executive leads, clinical directors, service directors, approved mental health professionals, and clinicians directly involved in delivering the service, visitor representatives and ED directors; A review of key relevant policy documents and other literature was undertaken to compose the framework • The evaluation was guided by six dimensions of healthcare quality: safe, timely, effective, efficient, equitable and person-centred
Dial House	Bagley, 2012 [22]	Social Return on Investment evaluation report	Interviews with stakeholders (visitors, carers, staff and external networks); visitors’ feedback survey and internal documents provided by the service	Fair	• For most visitors, Dial House is a place of sanctuary, a safe environment where they can relax and escape from the pressures that cause them to feel in crisis • Estimated that some alternative provision (potentially ED) would be needed in 75% of visits to Dial House • Estimated avoided suicides in 5% of visits • 50% of visitors attended on no more than three occasions, 30% of visitors made a few visits in most years, 12.5% of visitors used the service extensively in one year, 7.5% of visitors continue to use the service often, and become long term, frequent visitors • The service helps long-term visitors by preventing them from getting worse, helping them maintain their coping strategies and progressing to greater recovery
Dial House	Venner, 2009 [27]	Peer-reviewed article	Service manager’s narrative on the risk management approach at Dial House	Poor	• In 2008, 149 people made 1,051 visits (repeat visits) • Up to 75% of visitors to Dial House are suicidal. Self-injury was a presenting issue in up to 51% of visits then. The organisation is particularly effective for people who have been excluded from services or who have had difficulty engaging with services. Many visitors have violent or forensic histories, and many have been diagnosed with a personality disorder • According to visitors’ feedback, the five elements of effective support are: listening, ensuring visitors do not feel judged or assessed, treating people with warmth, kindness and respect, being in a different and calm environment, and peer support. Additionally, Dial House differs from other services because it’s more approachable, non-medical and there are no long waits • Visitors’ comments reinforce that the service helped them with avoiding ED and managing risk/preventing crises
Hillingdon Cove Crisis Café	Odlin, 2022 [23]	Updates report required by Health Board	Not specified	Poor	• General feedback from visitors was positive
St Vincent Safe Haven	PwC, 2018 [24]	Economic analysis of the service	Utilised visitor feedback survey provided by the service and ED data	Poor	• 37% of respondents would have gone to the ED if the café did not exist (suggested ED diversion) • 60% of respondents said the main reason for attending the service was to socialise with people like them • Visitors report a better care provision experience than ED, hospital admission or being alone without support • Total café benefits and cost savings exceed its cost on an ongoing basis (estimated annual saving of $33,860 from the café and cost savings of up to $225,400 through avoided mental health related ED presentations)
Safe Spaces	Nous Group, 2023 [25]	Service evaluation interim report	Utilised quantitative data on visitors provided by the service, relevant documents about the model and initial consults with broader stakeholders	Poor	• Most guests (86%) showed an improvement in distress levels between the start and the end of their Safe Space visit • Repeat visitors constitute most of the service activity: 32% of visitors and number of repeat visits ranging from 72–90% over the months • Allowing visitors to return to the Safe Spaces has enabled the avoidance of 176 visits to EDs, which reflects on $1.46 M estimated ED-related cost avoided • There is a need for clearer guidance on the service model, and to crystalise guidance and messaging on the target cohort without creating rigid exclusion criteria • Peer work is intensive and workforce structures need to accommodate for this. Expanded and standardised training and support are required
The Living Room	Heyland et al., 2013 [17]	Peer-reviewed article on the description of the service and initial findings	Visitor feedback survey provided by the service	Poor	• 93% ED deflection rate in the first year of operation (based on visitor feedback), representing estimated savings of $550,000 to the State of Illinois • On 84% of the occurrences in which visitors were deflected from EDs, they alleviated their crises sufficiently to decide to leave the service and return to the community • Most helpful interventions are the ability to talk through the situation and problem solve while at the space (80%); the coping skills (67%); the comfort of knowing the service was available during times of need (53%); and the resources/referrals given (40%)
The Living Room	Shattel et al., 2014 [26]	Peer-revied qualitative study on visitors’ experiences	Interviews with visitors and staff	Fair	• When compared to EDs, all visitors and staff said the service was as comfortable, safe, healing and a non-judgmental place • Staff reported that they care for each other and feel valued and supported • the study revealed confusion about what a mental health crisis is, and which types of emotional distress are most appropriate for TLR • Feelings of insecurity, loneliness, intimidation, fear, and discomfort with long wait hours characterised the experiences of EDs for persons in emotional distress • People in crises welcome care and support from registered nurses, counsellors, and peer workers in non-medical, safe, and comfortable settings
The Living Room	Heyland & Johnson, 2017 [29]	Peer-reviewed article/long-term evaluation of ED deflections	Follow-up phone call survey with visitors	Poor	• Long-term analysis showed an ED deflection rate of 94% after 30 days of visiting the service

**Table 3:** Summary of key findings

Service	Key Findings							
	Effective from the perspective of stakeholders	Safe environment from the perspective of stakeholders	Visitors felt listened to and treated with dignity and respect	Significant rates of repeat visits	Repeat visits associated with preventing crises and hospital avoidance	Avoided long waits at EDs	Estimated ED deflections	Better experience than ED
Aldershot Safe Haven Café [18, 19]	✔	✔	✔	✔	✔	✔	✔	✔
Community Crisis Intervention Service [20]	✔	✔	✔	?	?	✔	?	✔
Crisis Hubs [21]	✔	✔	✔	✔	✔	✔	✔	✔
Dial House [22, 27]	✔	✔	✔	✔	✔	✔	✔	✔
Hillingdon Cove Crisis Café [23]	✔	?	?	?	?	?	?	?
St Vincent Safe Haven Café [24]	✔	✔	✔	?	?	✔	✔	✔
Safe Spaces [25]	?	?	?	✔	✔	?	✔	?
The Living Room [17, 26, 29]	✔	✔	✔	?	?	✔	✔	✔

^a^ These findings are reported by the study authors and the methodological limitations across studies must be considered.

#### ED deflection

Eight publications reported on ED deflection (see Table 4) for six services [14, 15, 18, 19, 21–23]. Most services reported high rates of ED deflection and two of them also reported high rates of long-term deflection. However, most services had different definitions of ED deflection and how to measure them, as shown in Table 4, with the most common analyses coming from self-reports. Notably, many of these studies have suggested what could have been ED deflections while *assuming* the scenarios utilised [14, 15, 21]. Aldershot Safe Haven and St Vincent Safe Haven acknowledged in their study limitations that it would be difficult to attribute all changes in ED patterns entirely to the services because there may have been external factors influencing this and, without a control group, reductions in ED utilisation cannot definitively be attributed to the service [15].

**Table 4:** ED Deflection

Service	ED deflection analyses	Rate
Aldershot Safe Haven Café [18]	• Visitors reported through feedback survey when asked “If the Safe Haven had not been open today, where would you have looked for support?” they would have gone to ED	27%
	• Long-term deflection: evaluation analysed a cohort of visitors who had visited an ED at least once before the safe haven was launched. Their ED activity increased significantly three months before visiting the safe haven. Of these, around half (53%) had a decrease in ED attendance following their introduction to the service	53%
Crisis Hubs [21]	• Number of referrals that came from the ED	76% and 42% (two sites)
Dial House [22]	• Visitors reporting from feedback surveys they would have gone to ED if Dial House could not accommodate them and that they would have gone more frequently to ED as well	75%
St Vincent Safe Haven Café [24]	• Visitors that reported through feedback survey when asked “If the Safe Haven had not been open today, where would you have looked for support?” they would have gone to ED • Compared the average daily mental health ED presentations data from May to September 2018 (café operating months) to those presentations occurring during the same period in 2017 and then compared them with the same scenario occurring during the six months prior to the café opening. They observed that all sets of data reflected an increasing number of mental health ED presentations in the absence of the café compared to a decreasing trend of mental health ED presentations since the café’s opening. The final rate is an average from all scenarios.	12%
Safe Spaces [25]	• Repeat visitors - the report says that allowing guests to return to the Safe Space has enabled the avoidance of 176 visits to EDs. (does not specify how they reached the number of 176)	176 visits
The Living Room [17, 26]	• Number of visitors that left the service without the need to go to an ED	93%
	• Long-term deflection: phone call follow-up after 30 days of visiting TLR with 16 visitors, where 15 reported they no longer attended an ED after going to TLR	94%

^a^ These findings are reported by the authors and the methodological limitations across publications must be considered.

#### Recommendations

There were seven publications that provided recommendations [15, 17–19, 21–23]. The key recommendations as provided by each service are shown in the supplementary material (File 1, Online Resource 3). The most common ones were (1) extending the opening hours [15, 17, 19, 21], (2) enhancing service awareness [15, 17–19] and (3) increasing capacity [18, 19, 21]. Of particular note, Dial House reported that it refuses a large number of people yearly due to reaching full capacity. It was also noted that in some of these cases, the individuals used the ED instead, suggesting that these ED presentations could have been avoided if the service had more capacity. Therefore, increasing capacity might mean more ED deflections [19].

Additionally, it was recommended for some services that they improved accessibility [15, 18, 21], provided a more private space [15, 23], and in the Crisis Hubs specifically that staff had more person-centred training which, if possible, should involve people with lived experience [18]. In the Safe Spaces [22], it was found that training was not consistent among service providers for peer workers. The duration, intensity, and training format differed from online training modules to multi-day face-to-face, and access to one-to-one supervision sessions also varied. Therefore, it was recommended that a minimum standard training set for all staff was developed.

It was also indicated that it would be helpful to have a deeper understanding of the scope of the work and more guidance regarding service delivery criteria across all levels of the spaces. In The Living Room [23] and in the Safe Spaces [22] there was some confusion regarding who would be appropriate to use the services and it was, therefore, recommended that this be made clearer, while allowing for necessary flexibility in these guidelines [23]. Therefore, service providers were recommended to provide clearer service guidelines to promote consistency over time [22, 23].

## Discussion

This scoping review identified consistent characteristics and outcomes across the person-centred crisis support services examined. The methodological quality of most publications included is poor and therefore, outcomes should be considered with caution. These services are designed to be a safe alternative to EDs for people experiencing mental health crises. They offer one-to-one support, development of coping skills and safety plans, referrals to other services and programs if required, and a separate environment to the ED where visitors can drop in and not have to wait for long hours to receive care [14–24, 26]. Most of them are community-based crises interventions, but there are no differences reported in terms of philosophy of care or outcomes from the two hospital-based experiences or from the three Crisis Cafés, which suggests that these services can deliver the same results independently of where they are located or how they are designed if they maintain the same core features of person-centred care.

From the perspective of visitors and staff, these core features of person-centred care consist of providing comfortable, non-judgemental and compassionate safe spaces where visitors can reach out for help at an early point to prevent a crisis from escalating and to de-escalate immediate crisis while avoiding long waits at an intimidating, stigmatising and overstimulating environment such as an ED. The respectful approach by staff was one of the key elements to the services’ success - one of them specified that for some visitors, the peer workers are “the heart and soul” of the service [23]. Therefore, it is possible to conclude that the key ingredients to the successful experiences of person-centred care at crisis support services are engaging with respectful and compassionate staff in a safe and welcoming environment where there are no long waits and people can re-visit for support with immediate crises, ongoing distress and to prevent future crises without the need of hospital care. The perspective of broader stakeholders was only mentioned in the Crisis Hubs study [18] and suggested a different focus regarding effectiveness, which was associated with providing timely care and relieving pressure on ED staff. However, further research is needed to investigate the different perspectives across stakeholder levels.

Regarding these services’ outcomes, most visitors reported the services to be safe and effective across the reviewed publications. The proportion of this endorsement was however markedly lower at the Crisis Hubs than the other person-centred crisis support services. When examining the program characteristics of the service models to possibly explain this difference, the Crisis Hubs did not mention some of these core features in their report, such as having lived experience involvement or a non-medical environment. Additionally, it is the only service that has physicians as part of the staff. The shared characteristics across all models of person-centred crisis support services of not being located at an ED and providing access to more timely support are therefore suggested as the key features of effectiveness. This observation may also suggest that such services may be more effective when having lived experience involvement and be situated in a non-medical environment, since over a third of visitors reported not feeling safe at the Crisis Hubs. That said, the Crisis Hubs evaluation was the only one that had a framework co-designed in consultation with service stakeholders [25]. However, The Crisis Hubs report that presented the evaluation outcomes was assessed as poor quality for the purposes of this review. In contrast, the two peer-reviewed qualitative articles that focused on visitors’ experiences (and were assessed as fair quality) found that visitors welcome the support of mental health clinicians like nurses, psychologists and counsellors but feel more empowered and less intimidated by the fact that there are no physicians and that they can receive care in a non-medical environment [17, 23]. There is a clear need for high-quality evaluations that generate comparable data across different services to provide clarity about the best way for these services to be structured.

Other key findings were that half of the services reported significant rates of repeat visitors and there appears to be associations between repeat visits, crises prevention, and hospital avoidance [15, 16, 18, 19, 22, 24]. However, most publications did not touch specifically on repeat visitors or crisis prevention.

It is important to note that repeat visits and crises prevention are not explicitly mentioned as intended outcomes of crisis support services, and unclear model guidelines can create confusion regarding what is an appropriate focus for these services [22, 23]. However, one of the conclusions also made in The Living Room study was that it is essential to differentiate the definition of crisis and emergency to understand service appropriateness. A crisis can be defined as a state of feeling overwhelmed where individuals are unable to cope adequately with their life processes, while an emergency is the risk of harm to oneself and others; and if the crisis is not adequately addressed, it can escalate and lead to an emergency [23, 30]. A state of crisis and pre-emptive action to prevent a crisis may be hard to differentiate in the context of chronic distress. The study on The Living Room concluded that all types of crises should be welcomed in services like these [23]. The Safe Spaces evaluation had a similar conclusion, reporting that repeat visitors should be acknowledged as part of the model, as it is not feasible to establish a threshold for distress to define immediate crisis given the variety of ways that it can present [22].

In terms of ED diversion, only a few publications looked at estimating deflections [14, 15, 18, 19, 21, 26]. Although most of them showed high rates of ED deflections and long-term hospital avoidance, there was no consensus on a definition of ED deflection or on an approach to measure it. All the analyses were based on survey results, and some had additional ED data, which were mentioned as methodological limitations of the publications [15, 21].

Lastly, consistent recommendations arose across the publications reporting them [15, 17–19, 21, 23]. The main ones were increased opening hours and capacity and better service awareness, followed by accessibility, privacy, more training and clearer model guidelines. Therefore, recommendations for the model going forward are mostly associated with expanding the services’ functionalities and clarifying the scope of the work and service delivery rather than changing any of the core aspects of the model, which suggests that person-centred crisis support services are working as alternatives to EDs and may work better with further investment and clearer guidelines, according to the conclusions drawn from the publications included.

## Conclusions

This systematic scoping review aimed to describe the characteristics and outcomes found in the literature of person-centred crisis support services as alternatives to EDs. The services examined in this review are generally characterised by one-to-one support away from EDs where staff are more approachable and respectful, and visitors can drop in to receive more appropriate and timely care than at EDs. Key findings involved positive visitor feedback regarding person-centred treatment and environment, high rates of ED deflection and overlaps between repeat visits, crisis prevention and hospital avoidance. The main recommendations provided were increased opening hours and capacity and better service awareness and accessibility. However, the included publications have several methodological limitations according to their quality appraisal. As investment in these services within Australia expands, a pressing need emerges for high-quality evidence-based evaluation of the services, and so further research is needed to address these deficits. Robust evaluations containing evidence-based data on the implementation (what is being delivered and how), effectiveness, and economic aspects of the services would potentially support the expansion of these services. The establishment of this evidence would hold significant importance in shaping policymaking choices concerning crisis support services. It will also inform the future design, delivery and sustainability of the person-centred crisis support services and inform future research to help drive service improvements for people with lived experience of mental health crises.

## Electronic supplementary material

Below is the link to the electronic supplementary material.

Supplementary Material 1

Supplementary Material 2

## Data Availability

No datasets were generated or analysed during the current study.

## Footnotes

1 Mental health crises are referred to as such throughout this article as we understand that there is not a specific way to characterize a crisis presentation. These presentations may vary from social crises to acute crises that may or may not require medical care [1].
