# Supplementary material for: Person-centred crisis support services as alternatives to emergency departments: a systematic scoping review
Source: Soc Psychiatry Psychiatr Epidemiol. 2024 Oct 10;60(1):25–37. doi: 10.1007/s00127-024-02776-1 (PMC11790714; doi:10.1007/s00127-024-02776-1)
Supplement: Supplementary file 1 — Supplementary Material 1 [file 127_2024_2776_MOESM1_ESM.pdf]

## Supplementary Material – File 2

Quality Appraisal

**Article title:**

Person-centred crisis support services as alternatives to emergency departments: a systematic scoping review

**Journal name:**

Social Psychiatry and Psychiatric Epidemiology

**Authors:**

Danielle Postorivo[1,2], Stephen Parker[1,4], Harvey Whiteford[1,2,3], Zoe Papinczak[2,5], Zoe Rutherford[1,2]

**Affiliations:**

[1]School of Public Health, Faculty of Medicine, University of Queensland, Brisbane, QLD, Australia
[2]Queensland Centre for Mental Health Research, Brisbane, QLD, Australia
[3]Institute for Health Metrics and Evaluation, University of Washington, Seattle, Washington, USA
[4]Metro North Mental Health, Brisbane, QLD, Australia
[5]School of Human Movement and Nutrition Sciences, The University of Queensland, Brisbane, QLD, Australia

**Corresponding author:**

Danielle Postorivo

danielle.postorivo@uq.edu.au

# JBI CRITICAL APPRAISAL CHECKLIST FOR TEXT AND OPINION PAPERS

Author: **Vanessa Odlin** Year: **2022** Record: Mental Health Services: Cove Crisis Café, 16-25 Young Adult Mental Health And Crisis House

| | Yes | No | Unclear | Not applicable |
|---|---|---|---|---|
| **1. Is the source of the opinion clearly identified?**<br>P1 Source = Vanessa Odlin | X | ☐ | ☐ | ☐ |
| **2. Does the source of opinion have standing in the field of expertise?**<br>No information about the author is provided in the report. A review of the web identifies a LinkedIn profile that indicates that the author was the Director of Hillingdon and Mental Health (CNLW) at the time the report was written. | ☐ | ☐ | X | ☐ |
| **3. Are the interests of the relevant population the central focus of the opinion?** | X | ☐ | ☐ | ☐ |
| **4. Is the stated position the result of an analytical process, and is there logic in the opinion expressed?** | ☐ | ☐ | X | ☐ |
| **5. Is there reference to the extant literature?** | ☐ | X | ☐ | ☐ |
| **6. Is any incongruence with the literature/sources logically defended?** | ☐ | X | ☐ | ☐ |

Overall appraisal: Include X Exclude ☐ Seek further info ☐

Comments (Including reason for exclusion)

While information about stakeholder perspectives is provided in the form of testimonial statements, there is an absence of methodological detail that would be needed to support the trustworthiness of the analysis. This appears to reflect an internal report to the health service board. The level of detail and lack of a defined methodology in relation to work supports the allocation of the category of **POOR QUALITY** for the purposes of this scoping review.

# JBI CRITICAL APPRAISAL CHECKLIST FOR QUALITATIVE RESEARCH

Author: **Shattell et al.** Year: **2014** Record: A Recovery-Oriented Alternative to Hospital Emergency Departments for Persons in Emotional Distress: “The Living Room”

| | | Yes | No | Unclear | Not applicable |
|---|---|---|---|---|---|
| 1. | **Is there congruity between the stated philosophical perspective and the research methodology?** | X | ☐ | ☐ | ☐ |
| 2. | **Is there congruity between the research methodology and the research question or objectives?** | X | ☐ | ☐ | ☐ |
| 3. | **Is there congruity between the research methodology and the methods used to collect data?** | X | ☐ | ☐ | ☐ |
| 4. | **Is there congruity between the research methodology and the representation and analysis of data?**<br><br>Limited detail provided about the analytic process in the methods section.<br>Insufficient detail is available about the process through which illustrative transcript extracts were chosen.<br>Limited use of transcript extracts to establish adequate representation of the data.<br>No discussion of saturation. | ☐ | ☐ | X | ☐ |
| 5. | **Is there congruity between the research methodology and the interpretation of results?** | X | ☐ | ☐ | ☐ |
| 6. | **Is there a statement locating the researcher culturally or theoretically?**<br><br>Reflexivity issues not adequately considered. Researchers also described themselves as ‘community partners’ but this was not further explained. | ☐ | X | ☐ | ☐ |
| 7. | **Is the influence of the researcher on the research, and vice- versa, addressed?** | ☐ | ☐ | X | ☐ |
| 8. | **Are participants, and their voices, adequately represented?**<br><br>See 4. | ☐ | ☐ | X | ☐ |

**9. Is the research ethical according to current criteria or, for recent studies, and is there evidence of ethical approval by an appropriate body?** X ☐ ☐ ☐

**10. Do the conclusions drawn in the research report flow from the analysis, or interpretation, of the data?** X ☐ ☐ ☐

Acknowledgement of potential sources of bias is well considered.

Overall appraisal: Include X Exclude ☐ Seek further info ☐

Comments:

---

Overall rating is **FAIR QUALITY**. While there are limitations in the methodological description, the extent to which the source data is used to support the analysis, and consideration of reflexity, the authors consider the limitations of their work. A key consideration is the sampling approach and the possibility that recruitment from within the centre may have biased towards more favourable experiences.

© JBI, 2020. All rights reserved. JBI grants use of these tools for research purposes only. All other enquiries should be sent to jbisynthesis@adelaide.edu.au.

# JBI CRITICAL APPRAISAL CHECKLIST FOR TEXT AND OPINION PAPERS

Author: **North East Hampshire and Farnham CCG** Year: **2014** Record: 'The Safe Haven' Aldershot Evaluation Report July 2014

| | Yes | No | Unclear | Not applicable |
|---|---|---|---|---|
| 1. **Is the source of the opinion clearly identified?** | ☐ | X | ☐ | ☐ |
| 2. **Does the source of opinion have standing in the field of expertise?** | ☐ | ☐ | X | ☐ |
| 3. **Are the interests of the relevant population the central focus of the opinion?** | X | ☐ | ☐ | ☐ |
| 4. **Is the stated position the result of an analytical process, and is there logic in the opinion expressed?** While there is a logic to the process, this is rudimentary and inadequate to allow confidence in the assertions drawn.<br>Descriptive statistics presented in support of service evaluation. Also, a range of assertions are made about service impacts without supporting evidence being provided.<br>Survey data presented without consideration of response rates. No information about characteristics of survey completers/non-completers.<br>Data is presented on A&E attendances, despite these increasing for the first two months of the project (with a slight decrease in the third month) these are reported favourably. There is inadequate consideration of seasonal trends.<br>Cost effectiveness analyses are rudimentary.<br>Examples of feedback is presented without any defined methodology for how this has been sourced, analysed and chosen. | ☐ | ☐ | X | ☐ |
| 5. **Is there reference to the extant literature?** | ☐ | X | ☐ | ☐ |
| 6. **Is any incongruence with the literature/sources logically defended?** | ☐ | ☐ | ☐ | X |

Overall appraisal: Include X Exclude ☐ Seek further info ☐

Comment

Overall assessment of record quality is **POOR**. While evidence is presented this is not adequately supported by the limited available data.

# JBI CRITICAL APPRAISAL CHECKLIST FOR ECONOMIC EVALUATIONS

Author: **Heyland & Johnson** Year: **2017** Record: Evaluating an Alternative to the Emergency Department for Adults in Mental Health Crisis

| | | Yes | No | Unclear | Not applicable |
|---|---|---|---|---|---|
| 1. | **Is there a well-defined question?** | X | ☐ | ☐ | ☐ |
| 2. | **Is there a comprehensive description of alternatives?**<br>ED-based assessment is considered in general terms without consideration of what is locally available. Additionally, potential differences in the acuity of problems that can be assessed in an ED vs community setting are not considered. | ☐ | X | ☐ | ☐ |
| 3. | **Are all important and relevant costs and outcomes for each alternative identified?**<br>Consideration of costs is at a rudimentary level. | ☐ | X | ☐ | ☐ |
| 4. | **Has clinical effectiveness been established?**<br>While assertions are made about the effectiveness of the service this is not adequately established through the literature. | ☐ | X | ☐ | ☐ |
| 5. | **Are costs and outcomes measured accurately?**<br>Study relies on self-report of ED attendance rather than administrative data. Issues with sampling likely undermine representativeness. | ☐ | X | ☐ | ☐ |
| 6. | **Are costs and outcomes valued credibly?** | ☐ | ☐ | X | ☐ |
| 7. | **Are costs and outcomes adjusted for differential timing?** | ☐ | X | ☐ | ☐ |
| 8. | **Is there an incremental analysis of costs and consequences?** | ☐ | X | ☐ | ☐ |
| 9. | **Were sensitivity analyses conducted to investigate uncertainty in estimates of cost or consequences?** | ☐ | X | ☐ | ☐ |

| | | | | |
|---|---|---|---|---|
| **10. Do study results include all issues of concern to users?** | ☐ | X | ☐ | ☐ |
| **11. Are the results generalizable to the setting of interest in the review?** | ☐ | ☐ | X | ☐ |

Overall appraisal: Include X Exclude ☐ Seek further info ☐

Comments (Including reason for exclusion)

---

The Economic Evaluation checklist has been chosen as this was the strongest assertion made in the paper. There are issues with the adequacy of the method to support economic analysis. The article is **POOR QUALITY** with regards to informing the questions posed by the current review.

© JBI, 2020. All rights reserved. JBI grants use of these tools for research purposes only. All other enquiries should be sent to jbisynthesis@adelaide.edu.au.

# JBI CRITICAL APPRAISAL CHECKLIST FOR QUALITATIVE RESEARCH

Author: **Ennis and Walker** Year: **2022** Record: Experiences of a Community Crisis Intervention Service

| | | Yes | No | Unclear | Not applicable |
|---|---|---|---|---|---|
| 1. | **Is there congruity between the stated philosophical perspective and the research methodology?** | X | ☐ | ☐ | ☐ |
| 2. | **Is there congruity between the research methodology and the research question or objectives?** | X | ☐ | ☐ | ☐ |
| 3. | **Is there congruity between the research methodology and the methods used to collect data?** | X | ☐ | ☐ | ☐ |
| 4. | **Is there congruity between the research methodology and the representation and analysis of data?** Several transcript extracts are provided to demonstrate themes. | X | ☐ | ☐ | ☐ |
| 5. | **Is there congruity between the research methodology and the interpretation of results?** | X | ☐ | ☐ | ☐ |
| 6. | **Is there a statement locating the researcher culturally or theoretically?** Reflexivity issues do not appear to be well considered. | ☐ | X | ☐ | ☐ |
| 7. | **Is the influence of the researcher on the research, and vice- versa, addressed?** | ☐ | X | ☐ | ☐ |
| 8. | **Are participants, and their voices, adequately represented?** | X | ☐ | ☐ | ☐ |
| 9. | **Is the research ethical according to current criteria or, for recent studies, and is there evidence of ethical approval by an appropriate body?** No ethics statement in text. | ☐ | ☐ | X | ☐ |
| 10. | **Do the conclusions drawn in the research report flow from the analysis, or interpretation, of the data?** Yes, 5 interviews only | X | ☐ | ☐ | ☐ |

Overall appraisal: Include X Exclude ☐ Seek further info ☐

Comments (Including reason for exclusion)

Overall rating = **FAIR QUALITY**. Very small sample, issues of saturation not discussed (but suggested). Limited consideration of reflexity.

# JBI CRITICAL APPRAISAL CHECKLIST FOR TEXT AND OPINION PAPERS

Author: **Real-Improvement** Year: **2012** Record: Leeds Survivor Led Crisis Service: Full SROI Evaluation Report

| | Yes | No | Unclear | Not applicable |
|---|---|---|---|---|
| 1. **Is the source of the opinion clearly identified?** | X | ☐ | ☐ | ☐ |
| 2. **Does the source of opinion have standing in the field of expertise?** | ☐ | ☐ | X | ☐ |
| 3. **Are the interests of the relevant population the central focus of the opinion?** | X | ☐ | ☐ | ☐ |
| 4. **Is the stated position the result of an analytical process, and is there logic in the opinion expressed?** SROI is used as the primary approach, however additional data is also presented. Some key limitations in the available data are acknowledged. | X | ☐ | ☐ | ☐ |
| 5. **Is there reference to the extant literature?** | ☐ | X | ☐ | ☐ |
| 6. **Is any incongruence with the literature/sources logically defended?** | ☐ | ☐ | ☐ | X |

Overall appraisal: Include X Exclude ☐ Seek further info ☐

Comments (Including reason for exclusion)

While the economic analysis form may also be considered, this report goes beyond economic analyses. It is a comprehensive report, provides explicit logic for the method and acknowledges some key limitations in the data. Overall assessment is **FAIR QUALITY** with the qualification that it reflects a grey literature report.

# JBI CRITICAL APPRAISAL CHECKLIST FOR TEXT AND OPINION PAPERS

Author: **Health Innovation Network** Year: **2022** Record: Evaluating NHS Mental Health Crisis Hubs in London: Final Report

| | Yes | No | Unclear | Not applicable |
|---|---|---|---|---|
| **1. Is the source of the opinion clearly identified?** Report produced by a consultancy group but with clear documentation of extensive stakeholder consultation. | X | ☐ | ☐ | ☐ |
| **2. Does the source of opinion have standing in the field of expertise?** | ☐ | ☐ | X | ☐ |
| **3. Are the interests of the relevant population the central focus of the opinion?** | X | ☐ | ☐ | ☐ |
| **4. Is the stated position the result of an analytical process, and is there logic in the opinion expressed?** Mixed quantitative and qualitative data is presented to support the conclusions drawn. Limited description of methodology through which some data was derived and its completeness. Lack of information about how extracts were chosen. Statistical data on outcomes presented in Table 10 do not appear to reflect an appropriate statistical test (use of t-test for categorical data). | ☐ | X | ☐ | ☐ |
| **5. Is there reference to the extant literature?** | ☐ | X | ☐ | ☐ |
| **6. Is any incongruence with the literature/sources logically defended?** | ☐ | ☐ | ☐ | X |

Overall appraisal: Include X Exclude ☐ Seek further info ☐

Comments (Including reason for exclusion)

Overall assessment is **POOR QUALITY**. The strength of this report is the apparent breadth of stakeholder consultation and consideration of broadly relevant issues. The lack of adequate methodological description undermines the trustworthiness of the qualitative data presented. Also, there are issues with the adequacy of quantitative data presented.

# JBI CRITICAL APPRAISAL CHECKLIST FOR TEXT AND OPINION PAPERS

Author: **Nous Group** Year: **2023** Record: Safe Spaces Evaluation Progress Report

| | Yes | No | Unclear | Not applicable |
|---|---|---|---|---|
| 1. **Is the source of the opinion clearly identified?** | X | ☐ | ☐ | ☐ |
| 2. **Does the source of opinion have standing in the field of expertise?** Known high-level management consultancy firm who has produced commissioned reports in the Australian context. | X | ☐ | ☐ | ☐ |
| 3. **Are the interests of the relevant population the central focus of the opinion?** | X | ☐ | ☐ | ☐ |
| 4. **Is the stated position the result of an analytical process, and is there logic in the opinion expressed?** Well-presented and clear assertions. However, the extent to which these are grounded in relevant data is not always clear. Limited methodological description (apart from quantitative analyses). Reliance on secondary data. | ☐ | ☐ | X | ☐ |
| 5. **Is there reference to the extant literature?** | ☐ | X | ☐ | ☐ |
| 6. **Is any incongruence with the literature/sources logically defended?** | ☐ | ☐ | ☐ | X |

Overall appraisal: Include X Exclude ☐ Seek further info ☐

Comments (Including reason for exclusion)

Presentation of SUDS data is a relative strength, including statistical analyses and completion of regression analyses to explore this further. A range of assertions and case studies are presented without adequately describing the process through which these were generated – this undermines trustworthiness. Based on the information presented, this record was rated as **POOR QUALITY** for the purposes of informing the scoping review.

# JBI CRITICAL APPRAISAL CHECKLIST FOR QUALITATIVE RESEARCH

Author: **Venner** Year: **2009** Record: Risk management in a survivor-led crisis service

| | Yes | No | Unclear | Not applicable |
|---|---|---|---|---|
| 1. **Is there congruity between the stated philosophical perspective and the research methodology?** | ☐ | ☐ | ☐ | X |
| 2. **Is there congruity between the research methodology and the research question or objectives?** | ☐ | ☐ | ☐ | X |
| 3. **Is there congruity between the research methodology and the methods used to collect data?** | ☐ | ☐ | ☐ | X |
| 4. **Is there congruity between the research methodology and the representation and analysis of data?** | ☐ | ☐ | ☐ | X |
| 5. **Is there congruity between the research methodology and the interpretation of results?** | ☐ | ☐ | ☐ | X |
| 6. **Is there a statement locating the researcher culturally or theoretically?** | X | ☐ | ☐ | ☐ |
| 7. **Is the influence of the researcher on the research, and vice- versa, addressed?** | ☐ | X | ☐ | ☐ |
| 8. **Are participants, and their voices, adequately represented?** | ☐ | ☐ | X | ☐ |
| 9. **Is the research ethical according to current criteria or, for recent studies, and is there evidence of ethical approval by an appropriate body?** | ☐ | X | ☐ | ☐ |
| 10. **Do the conclusions drawn in the research report flow from the analysis, or interpretation, of the data?** | ☐ | ☐ | X | ☐ |

Overall appraisal: Include X Exclude ☐ Seek further info ☐

Comments (Including reason for exclusion)

This paper presents qualitative data but does not describe the analytic method. As such it is has been categorised as **POOR QUALITY** evidence for the purposes of the current review.

# JBI CRITICAL APPRAISAL CHECKLIST FOR TEXT AND OPINION PAPERS

Author: **Wessex Academic Health Science Network** Year: **2017** Record: Independent Evaluation of the North East Hampshire and Farnham Vanguard Aldershot Safe Haven Service

| | Yes | No | Unclear | Not applicable |
|---|---|---|---|---|
| **1. Is the source of the opinion clearly identified?** | X | ☐ | ☐ | ☐ |
| **2. Does the source of opinion have standing in the field of expertise?** | ☐ | ☐ | X | ☐ |
| **3. Are the interests of the relevant population the central focus of the opinion?** | X | ☐ | ☐ | ☐ |
| **4. Is the stated position the result of an analytical process, and is there logic in the opinion expressed?** | ☐ | ☐ | X | ☐ |
| **5. Is there reference to the extant literature?** | ☐ | X | ☐ | ☐ |
| **6. Is any incongruence with the literature/sources logically defended?** | ☐ | ☐ | ☐ | X |

Overall appraisal: Include X Exclude ☐ Seek further info ☐

**Comments (Including reason for exclusion)**

Trend-based quantitative analyses.

Reliance on self-report for some of the service attendance data.

IPad survey data was based on 79 respondents only, no information is available to establish the likely representativeness of these participants.

The qualitative methodology for the analysis of free-text responses is not well described. However, the table provides comprehensive detail of extracts supporting the themes identified.

The limitations in methodological description in relation to the qualitative data, as well as the trend-based analyses for quantitative data is why this record has been categorised as **POOR QUALITY** for the purposes of this review.

# JBI CRITICAL APPRAISAL CHECKLIST FOR ECONOMIC EVALUATIONS

Author: **PWC** Year: **2018** Record: Economic impact of the Safe Haven Café Melbourne

| | Yes | No | Unclear | Not applicable |
|---|---|---|---|---|
| **1. Is there a well-defined question?** | X | ☐ | ☐ | ☐ |
| **2. Is there comprehensive description of alternatives?** | ☐ | X | ☐ | ☐ |
| **3. Are all important and relevant costs and outcomes for each alternative identified?** | ☐ | ☐ | X | ☐ |
| **4. Has clinical effectiveness been established?** | ☐ | X | ☐ | ☐ |
| **5. Are costs and outcomes measured accurately?** | ☐ | ☐ | X | ☐ |
| **6. Are costs and outcomes valued credibly?** | ☐ | ☐ | X | ☐ |
| **7. Are costs and outcomes adjusted for differential timing?** | ☐ | X | ☐ | ☐ |
| **8. Is there an incremental analysis of costs and consequences?** | ☐ | X | ☐ | ☐ |
| **9. Were sensitivity analyses conducted to investigate uncertainty in estimates of cost or consequences?** | ☐ | X | ☐ | ☐ |
| **10. Do study results include all issues of concern to users?** | ☐ | X | ☐ | ☐ |
| **11. Are the results generalizable to the setting of interest in the review?** | X | ☐ | ☐ | ☐ |

Overall appraisal: Include X Exclude ☐ Seek further info ☐

**Comments (Including reason for exclusion)**

This grey literature report presents a commissioned economic analysis of an Australian crisis support service. A key concern in the report is the identification of the risk of bias in the measurement of ED diversion based on self-report but choosing to focus on this in the key findings. This occurred despite validation efforts looking at administrative data suggesting it was an overestimate. Furthermore, there is a lack of complexity in the analysis to support inferences drawn from trend-based assessments of service effectiveness. Given these issues I have rated the report as **POOR QUALITY** for the purposes of the review.

# JBI CRITICAL APPRAISAL CHECKLIST FOR TEXT AND OPINION PAPERS

Author: **Heyland, Emery, Shattell** Year: **2013** Record: The Living Room, a Community Crisis Respite Program: Offering People in Crisis an Alternative to Emergency Departments

| | Yes | No | Unclear | Not applicable |
|---|---|---|---|---|
| 1. Is the source of the opinion clearly identified? | X | ☐ | ☐ | ☐ |
| 2. Does the source of opinion have standing in the field of expertise? | ☐ | ☐ | X | ☐ |
| 3. Are the interests of the relevant population the central focus of the opinion? | X | ☐ | ☐ | ☐ |
| 4. Is the stated position the result of an analytical process, and is there logic in the opinion expressed? | ☐ | ☐ | X | ☐ |
| 5. Is there reference to the extant literature? | X | ☐ | ☐ | ☐ |
| 6. Is any incongruence with the literature/sources logically defended? | ☐ | X | ☐ | ☐ |

Overall appraisal: Include X Exclude ☐ Seek further info ☐

Comments (Including reason for exclusion)

This record fits under the text and opinion category rather than the qualitative, as it presents service use data, makes assertions about cost-effectiveness, and presents anecdotal reports of service visitors. There is no defined methodology. There are limitations associated with the assumed level of ED diversion. Based on these issues, this record was categorised as **POOR QUALITY** for the purposes of the review.

# Critical Appraisal tools for use in JBI Systematic Reviews

jbi.global

CRICOS Provider Number 00123M

# JBI CRITICAL APPRAISAL CHECKLIST FOR ECONOMIC EVALUATIONS

*How to cite: Gomersall JS, Jadotte YT, Xue Y, Lockwood S, Riddle D, Preda A. Conducting systematic reviews of economic evaluations. Int J Evid Based Healthc. 2015;13(3):170–178.*

This tool is informed by the work of Drummond et al, Methods for the economic evaluation of health care programmes. 2nd Edition. Oxford: Oxford Medical Publications, 1997.

### 1. Is there a well-defined question/objective?

Consider the following before marking the study as compliant with this quality criterion:

- Is the objective/question of the study clearly stated?
- Does the statement reflect the perspective (e.g. patient or community or societal or health provider) used in measurement of costs or/and cost effectiveness?
- Was the study placed in a particular decision making context?

### 2. Is there a comprehensive description of alternatives?

To be marked as compliant with this criterion the authors of the study should offer a clear description of the intervention or interventions considered in the economic evaluation and the comparator or comparators. Compliance does not require that a broad range of interventions and comparators was considered. What is important here is clear description of the nature of the intervention and comparator whose cost/effeteness was measured.

### 3. Are all important and relevant costs and outcomes for each alternative identified?

This quality criterion assesses the comprehensiveness and relevant of the cost and cost effectiveness outcomes measured in the economic evaluation. When deciding whether all important costs and outcomes have been identified/measured in the study reflect on whether the outcomes are sufficient in light of the objectives of the study. It is appropriate for a study that has the objective of measuring a narrow range of costs and benefits to identify and measure a limited range. However, the limits of the narrow approach should be drawn out in the study. It is not appropriate for a study which implies in its objective statement that it measures a broad range of costs for a broad range out outcomes to include only a very limited range of relevant costs and outcomes.

### 4. Has clinical effectiveness been established?

To assess compliance with this quality criterion requires considering whether the study has reported the evidence used to derive the effectiveness estimate and the level of this evidence. If it is not clear how the effectiveness estimate was derived, the study cannot be marked as compliant. To achieve compliance for this criterion the effectiveness estimate in the evaluation does not need to be derived from the same study as the resource use/cost estimate. What is important is the there is a solid evidence base under-pinning the assumptions about the direction and magnitude of the effectiveness measure(s) used in the evaluation.

### 5. Are costs and outcomes measured accurately?

This quality criterion assesses whether the study has used appropriate/best practice measurement method to measure costs and effectiveness. To decide whether a study should be marked as compliant consider whether the methods section of the paper offers a detail description of the measures used for costs and outcomes and how it justifies them. In addition, consider whether the authors/study implementers discussed any limitations associated with the measures used and concerns about the accuracy of measurement. In economic evaluations it is often difficult to

© JBI, 2020. All rights reserved. JBI grants use of these tools for research purposes only. All other enquiries should be sent to jbisynthesis@adelaide.edu.au.

measure costs and outcomes accurately, and hence in many cases this quality criterion will be difficult to achieve.

6. **Are costs and outcomes valued credibly?**

   This quality criterion assesses whether appropriate prices were used to value costs and the validity of the valuation of benefits. It requires considering the method description and judging where there is a sufficient explanation about how costs and outcomes were valued and whether the justification for it is persuasive.

7. **Are costs and outcomes adjusted for differential timing?**

   To be marked compliant for this question the study should have identified and justified the discount rate used. The time frame over which the study was conducted should also have been identified and justified.

8. **Is there any incremental analysis of costs and consequences?**

   To achieve compliance the paper should report a measure that shows the change in costs and benefits for the intervention and comparator for a marginal shift in resources from the comparator to the intervention.

9. **Were sensitivity analysis conducted to investigate uncertainty in estimates of costs or outcomes?**

   Sensitivity analysis is critical for establishing the validity of any economic evaluations results. To be compliant a study must present sensitivity testing results that describe how the study findings vary with changes in key variables (for example relative prices, and intervention estimates? conducted to check the robustness of findings.

10. **Do study results include all issues of concern to users?**

    This question reflects on the comprehensiveness of coverage in the reporting of results. In deciding whether to mark the study as compliance consider whether the range of measures presented provider answers to all the questions users/decision makers would want to know when taking a decision about whether to implement the program examined (or cutting it)?

11. **Are the results generalizable to the setting of interest in the review?**

    To be marked as compliant for this last quality criterion the paper should: (i) have described the study setting adequately; (ii) discuss the issue of transferability of findings and how the results are generalizable to other settings with similar characteristics

© JBI, 2020. All rights reserved. JBI grants use of these tools for research purposes only. All other enquiries should be sent to jbisynthesis@adelaide.edu.au.

# JBI CRITICAL APPRAISAL CHECKLIST FOR QUALITATIVE RESEARCH

*How to cite: Lockwood C, Munn Z, Porritt K. Qualitative research synthesis: methodological guidance for systematic reviewers utilizing meta-aggregation. Int J Evid Based Healthc. 2015;13(3):179–187.*

1. **Congruity between the stated philosophical perspective and the research methodology**

   Does the report clearly state the philosophical or theoretical premises on which the study is based? Does the report clearly state the methodological approach adopted on which the study is based? Is there congruence between the two? For example:

   A report may state that the study adopted a critical perspective and participatory action research methodology was followed. Here there is congruence between a critical view (focusing on knowledge arising out of critique, action and reflection) and action research (an approach that focuses on firstly working with groups to reflect on issues or practices, then considering how they could be different; then acting to create a change; and finally identifying new knowledge arising out of the action taken). However, a report may state that the study adopted an interpretive perspective and used survey methodology. Here there is incongruence between an interpretive view (focusing on knowledge arising out of studying what phenomena mean to individuals or groups) and surveys (an approach that focuses on asking standard questions to a defined study population); a report may state that the study was qualitative or used qualitative methodology (such statements do not demonstrate rigour in design) or make no statement on philosophical orientation or methodology.

2. **Congruity between the research methodology and the research question or objectives**

   Is the study methodology appropriate for addressing the research question? For example:
   A report may state that the research question was to seek understandings of the meaning of pain in a group of people with rheumatoid arthritis and that a phenomenological approach was taken. Here, there is congruity between this question and the methodology. A report may state that the research question was to establish the effects of counselling on the severity of pain experience and that an ethnographic approach was pursued. A question that tries to establish cause-and effect cannot be addressed by using an ethnographic approach (as ethnography sets out to develop understandings of cultural practices) and thus, this would be incongruent.

© JBI, 2020. All rights reserved. JBI grants use of these tools for research purposes only. All other enquiries should be sent to jbisynthesis@adelaide.edu.au.

3. **Congruity between the research methodology and the methods used to collect data**

   Are the data collection methods appropriate to the methodology? For example:

   A report may state that the study pursued a phenomenological approach and data was collected through phenomenological interviews. There is congruence between the methodology and data collection; a report may state that the study pursued a phenomenological approach and data was collected through a postal questionnaire. There is incongruence between the methodology and data collection here as phenomenology seeks to elicit rich descriptions of the experience of a phenomena that cannot be achieved through seeking written responses to standardized questions.

4. **Congruity between the research methodology and the representation and analysis of data**

   Are the data analyzed and represented in ways that are congruent with the stated methodological position? For example:

   A report may state that the study pursued a phenomenological approach to explore people's experience of grief by asking participants to describe their experiences of grief. If the text generated from asking these questions is searched to establish the meaning of grief to participants, and the meanings of all participants are included in the report findings, then this represents congruity; the same report may, however, focus only on those meanings that were common to all participants and discard single reported meanings. This would not be appropriate in phenomenological work.

5. **There is congruence between the research methodology and the interpretation of results**

   Are the results interpreted in ways that are appropriate to the methodology? For example:

   A report may state that the study pursued a phenomenological approach to explore people's experience of facial disfigurement and the results are used to inform practitioners about accommodating individual differences in care. There is congruence between the methodology and this approach to interpretation; a report may state that the study pursued a phenomenological approach to explore people's experience of facial disfigurement and the results are used to generate practice checklists for assessment. There is incongruence between the methodology and this approach to interpretation as phenomenology seeks to understand the meaning of a phenomenon for the study participants and cannot be interpreted to suggest that this can be generalized to total populations to a degree where standardized assessments will have relevance across a population.

© JBI, 2020. All rights reserved. JBI grants use of these tools for research purposes only. All other enquiries should be sent to jbisynthesis@adelaide.edu.au.

6. **Locating the researcher culturally or theoretically**

   Are the beliefs and values, and their potential influence on the study declared? For example:

   The researcher plays a substantial role in the qualitative research process and it is important, in appraising evidence that is generated in this way, to know the researcher's cultural and theoretical orientation. A high quality report will include a statement that clarifies this.

7. **Influence of the researcher on the research, and vice-versa, is addressed**

   Is the potential for the researcher to influence the study and for the potential of the research process itself to influence the researcher and her/his interpretations acknowledged and addressed? For example:

   Is the relationship between the researcher and the study participants addressed? Does the researcher critically examine her/his own role and potential influence during data collection? Is it reported how the researcher responded to events that arose during the study?

8. **Representation of participants and their voices**

   Generally, reports should provide illustrations from the data to show the basis of their conclusions and to ensure that participants are represented in the report.

9. **Ethical approval by an appropriate body**

   A statement on the ethical approval process followed should be in the report.

10. **Relationship of conclusions to analysis, or interpretation of the data**

    This criterion concerns the relationship between the findings reported and the views or words of study participants. In appraising a paper, appraisers seek to satisfy themselves that the conclusions drawn by the research are based on the data collected; data being the text generated through observation, interviews or other processes.

© JBI, 2020. All rights reserved. JBI grants use of these tools for research purposes only. All other enquiries should be sent to jbisynthesis@adelaide.edu.au.

# JBI CRITICAL APPRAISAL CHECKLIST FOR TEXT AND OPINION

How to cite: *McArthur A, Klugarova J, Yan H, Florescu S. Innovations in the systematic review of text and opinion. Int J Evid Based Healthc. 2015;13(3):188–195.*

Answers: Yes, No, Unclear or Not/Applicable

1. **Is the source of the opinion clearly identified?**

   Is there a named author? Unnamed editorial pieces in journals or newspapers, or magazines give broader licence for comment, however authorship should be identifiable.

2. **Does the source of opinion have standing in the field of expertise?**

   The qualifications, current appointment and current affiliations with specific groups need to be stated in the publication and the reviewer needs to be satisfied that the author(s) has some standing within the field.

3. **Are the interests of the relevant population the central focus of the opinion?**

   The aim of this question is to establish the author's purpose in writing the paper by considering the intended audience. If the review topic is related to a clinical intervention, or aspect of health care delivery, a focus on health outcomes will be pertinent to the review. However, if for example the review is focused on addressing an issue of inter-professional behaviour or power relations, a focus on the relevant groups is desired and applicable. Therefore this question should be answered in context with the purpose of the review.

4. **Is the stated position the result of an analytical process, and is there logic in the opinion expressed?**

   In order to establish the clarity or otherwise of the rationale or basis for the opinion, give consideration to the direction of the main lines of argument. Questions to pose of each textual paper include: What are the main points in the conclusions or recommendations? What arguments does the author use to support the main points? Is the argument logical? Have important terms been clearly defined? Do the arguments support the main points?

5. **Is there reference to the extant literature?**

   If there is reference to the extant literature, is it a non-biased, inclusive representation, or is it a non-critical description of content specifically supportive of the line of argument being put forward? These considerations will highlight the robustness of how cited literature was managed.

6. **Is any incongruence with the literature/sources logically defended?**

   Is there any reference provided in the text to ascertain if the opinion expressed has wider support? Consider also if the author demonstrated awareness of alternate or dominant opinions in the literature and provided an informed defence of their position as it relates to other or similar discourses.

© JBI, 2020. All rights reserved. JBI grants use of these tools for research purposes only. All other enquiries should be sent to jbisynthesis@adelaide.edu.au.
