# Supplementary material for: Person-centred crisis support services as alternatives to emergency departments: a systematic scoping review
Source: Soc Psychiatry Psychiatr Epidemiol. 2024 Oct 10;60(1):25–37. doi: 10.1007/s00127-024-02776-1 (PMC11790714; doi:10.1007/s00127-024-02776-1)
Supplement: Supplementary file 2 — Supplementary Material 2 [file 127_2024_2776_MOESM2_ESM.pdf]

Supplementary Material – File 1

PRISMA-ScR checklist, search terms and key recommendations

Article title:

Person-centred crisis support services as alternatives to emergency departments: a systematic scoping review

Journal name:

Social Psychiatry and Psychiatric Epidemiology

Authors:

Danielle Postorivo[1,2], Stephen Parker[1,4], Harvey Whiteford[1,2,3], Zoe Papinczak[2,5], Zoe Rutherford[1,2]

Affiliations:

[1]School of Public Health, Faculty of Medicine, University of Queensland, Brisbane, QLD, Australia
[2]Queensland Centre for Mental Health Research, Brisbane, QLD, Australia
[3]Institute for Health Metrics and Evaluation, University of Washington, Seattle, Washington, USA
[4]Metro North Mental Health, Brisbane, QLD, Australia
[5]School of Human Movement and Nutrition Sciences, The University of Queensland, Brisbane, QLD, Australia

Corresponding author:

Danielle Postorivo

danielle.postorivo@uq.edu.au

Online Resource 1. Preferred Reporting Items for Systematic Reviews and Meta-Analysis Extension for Scoping Reviews (PRISMA-ScR) checklist

SECTION	ITEM	PRISMA-ScR CHECKLIST ITEM	REPORTED ON PAGE #
**TITLE**			
Title	1	Identify the report as a scoping review.	1
**ABSTRACT**			
Structured summary	2	Provide a structured summary that includes (as applicable): background, objectives, eligibility criteria, sources of evidence, charting methods, results, and conclusions that relate to the review questions and objectives.	1
**INTRODUCTION**			
Rationale	3	Describe the rationale for the review in the context of what is already known. Explain why the review questions/objectives lend themselves to a scoping review approach.	2
Objectives	4	Provide an explicit statement of the questions and objectives being addressed with reference to their key elements (e.g., population or participants, concepts, and context) or other relevant key elements used to conceptualize the review questions and/or objectives.	3
**METHODS**			
Protocol and registration	5	Indicate whether a review protocol exists; state if and where it can be accessed (e.g., a Web address); and if available, provide registration information, including the registration number.	3
Eligibility criteria	6	Specify characteristics of the sources of evidence used as eligibility criteria (e.g., years considered, language, and publication status), and provide a rationale.	4
Information sources*	7	Describe all information sources in the search (e.g., databases with dates of coverage and contact with authors to identify additional sources), as well as the date the most recent search was executed.	3
Search	8	Present the full electronic search strategy for at least 1 database, including any limits used, such that it could be repeated.	3
Selection of sources of evidence†	9	State the process for selecting sources of evidence (i.e., screening and eligibility) included in the scoping review.	3
Data charting process‡	10	Describe the methods of charting data from the included sources of evidence (e.g., calibrated forms or forms that have been tested by the team before their use, and whether data charting was done independently or in duplicate) and any	4

SECTION	ITEM	PRISMA-ScR CHECKLIST ITEM	REPORTED ON PAGE #
		processes for obtaining and confirming data from investigators.	
Data items	11	List and define all variables for which data were sought and any assumptions and simplifications made.	4
Critical appraisal of individual sources of evidence§	12	If done, provide a rationale for conducting a critical appraisal of included sources of evidence; describe the methods used and how this information was used in any data synthesis (if appropriate).	5
Synthesis of results	13	Describe the methods of handling and summarizing the data that were charted.	4
**RESULTS**			
Selection of sources of evidence	14	Give numbers of sources of evidence screened, assessed for eligibility, and included in the review, with reasons for exclusions at each stage, ideally using a flow diagram.	4
Characteristics of sources of evidence	15	For each source of evidence, present characteristics for which data were charted and provide the citations.	3
Critical appraisal within sources of evidence	16	If done, present data on critical appraisal of included sources of evidence (see item 12).	5
Results of individual sources of evidence	17	For each included source of evidence, present the relevant data that were charted that relate to the review questions and objectives.	6
Synthesis of results	18	Summarize and/or present the charting results as they relate to the review questions and objectives.	11
**DISCUSSION**			
Summary of evidence	19	Summarize the main results (including an overview of concepts, themes, and types of evidence available), link to the review questions and objectives, and consider the relevance to key groups.	22
Limitations	20	Discuss the limitations of the scoping review process.	24
Conclusions	21	Provide a general interpretation of the results with respect to the review questions and objectives, as well as potential implications and/or next steps.	24
**FUNDING**			
Funding	22	Describe sources of funding for the included sources of evidence, as well as sources of funding for the scoping review. Describe the role of the funders of the scoping review.	25

From: Tricco AC, Lillie E, Zarin W, O'Brien KK, Colquhoun H, Levac D, et al. PRISMA Extension for Scoping Reviews (PRISMAScR): Checklist and Explanation. Ann Intern Med. 2018;169:467–473. doi: 10.7326/M18-0850.

Online resource 2. Search terms used in electronic databases

**Concept 1**	Mental health crisis service OR crisis support service OR safe space OR safe place OR safe haven cafe OR crisis intervention OR crisis service OR crisis response
**Concept 2**	person-centred OR recovery-oriented OR recovery OR peer-run OR peer-led OR peer support OR non-clinical
**Concept 3**	alternative to emergency departments OR hospital diversion OR hospital avoidance OR crisis alternatives
**Concept 4**	evaluation OR evaluation report OR effectiveness

[a] The search strategy was adapted for each included database

[b] To explore documents available in the grey literature and limit the number of records to be screened, a targeted advanced search technique limiting results to documents was used on Google and Google Scholar to identify Word and PDF files only. This was done by adding the suffixes filetype:pdf and filetype:word to the search string.

Online resource 3. Recommendations

Service	Key Recommendations						
	Extended opening hours	More staff	Better service awareness	Better accessibility	Training	Privacy	Clearer model guidelines
Aldershot Safe Haven Café	✓	?	✓	✓	-	✓	?
Community Crisis Intervention Service	✓	?	✓	-	-	-	?
Crisis Hubs	-	✓	✓	✓	✓	-	?
Dial House	✓	✓	✓	-	-	-	?
St Vincent Safe Haven Café	✓	✓	✓	✓	-	-	?
Safe Spaces	✓	?	?	✓	✓	-	✓
The Living Room	?	?	?	?	-	✓	✓
